# Four-Dimensional Scaling of Dipole Polarizability: From Single-Particle Models to Atoms and Molecules

**DOI:** 10.1021/acs.jctc.4c00582

**Published:** 2024-07-17

**Authors:** Szabolcs Góger, Mohammad Reza Karimpour, Alexandre Tkatchenko

**Affiliations:** Department of Physics and Materials Science, University of Luxembourg, L-1511 Luxembourg City, Luxembourg

## Abstract

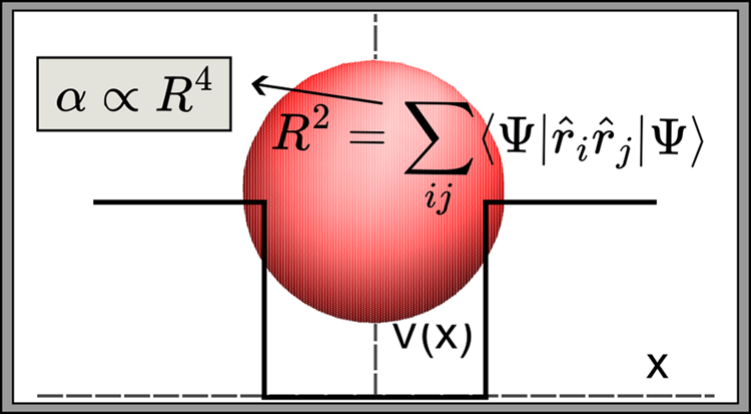

Scaling laws enable the determination of physicochemical properties of molecules and materials as a function of their size, density, number of electrons or other easily accessible descriptors. Such relations can be counterintuitive and nonlinear, and ultimately yield much needed insight into quantum mechanics of many-particle systems. In this work, we show on the basis of single-particle models, multielectron atoms and molecules that the dipole polarizability of quantum systems is generally proportional to the fourth power of a characteristic length, computed from the ground-state wave function. This four-dimensional (4D) scaling is independent of the ratio of bound-to-bound and bound-to-continuum electronic transitions and applies to many-electron atoms when a correlated length metric is used. Finally, this scaling law is applied to predict the polarizability of molecules by electrostatically coupled atoms-in-molecules approach, obtaining approximately 8% absolute and relative accuracy with respect to hybrid density functional theory (DFT) on the QM7–X data set of organic molecules, providing an efficient and scalable model for the anisotropic polarizability tensors of extended (bio)molecules.

## 1 Introduction

Predicting physicochemical properties of many-electron systems (including molecules, materials, or interfaces) from simple descriptors, such as molecular geometry or number of electrons, is a fundamental task for understanding quantum mechanics and for the rational design of matter with desired properties. Although accurate *ab initio* methods and molecular dynamics simulations can be done explicitly for each system at hand, their unfavorable scaling hinders applications to large data sets or extended systems. Scaling laws aim to capture the connection between the fundamental features of molecular systems and their physicochemical properties, providing a simple and general complementary approach to accurate calculation methods. Such relations are readily utilized, among other fields, in polymer science, response function theory, atomic and molecular spectroscopy, and conceptual density functional theory (DFT).^1−7^

Scaling laws at molecular or mesoscopic scales can be highly nonlinear and nontrivial due to quantum-mechanical and collective effects. The dispersion coefficients of nanostructures can change nontrivially with the system size due to collective quantum fluctuations,^8^ and the magnitude and sign of the power scaling exponent explictly depend on the chemical structure. The design of molecules for specific nonlinear optical applications relies on understanding the scaling of electromagnetic properties with molecular size, often studied case by case for molecules with different functionalities.^4^ In plasma physics, specifically when studying highly excited Rydberg atoms, most practical observables are expressed using scaling laws of the excitation number.^6^ Conceptual density functional theory also extensively utilizes scaling relations between different molecular descriptors such as softness, Fukui function, ionization potential, and electron affinity.^7^ In particular, the connection between size, orbital energies, polarizability and chemical properties form the basis of the hard/soft acid/base (HSAB) theory, being a key ingredient to chemical intuition.^9^

The scaling behavior of dipole polarizability plays a major role in the description of intermolecular interactions, as well as electron correlation, chemical bonding, protein folding, and bulk phase characteristics,^10−15^ and exhibits nontrivial scaling laws. Based on a model of a conducting sphere, classical polarizability can be shown to scale with the volume of the system, i.e., α = (4πϵ_0_)*R*_cl_^3^, where ϵ_0_ is the vacuum permittivity and *R*_cl_ is the radius of a conducting spherical shell.^16^ Alternatively, the size of atomic systems can be characterized by the van der Waals radius, with a proportionality of the form α ∝ *R*_vdW_^7^ found by studying two coupled quantum Drude oscillators.^17^ Originally proposed for confined quantum-mechanical systems,^18^ a four-dimensional (4D) scaling law (with effective mass μ and effective charge *q*) i.e., α ∝ *R*_quantum_^4^ was shown to hold for a wide range of quantum-mechanical model systems^19^ (for the definition of *R*_quantum_, see Section 2). Curiously, this four-dimensional scaling resembles the connection between electron density and exchange energy in a homogeneous electron gas,^20^ i.e., *E*_*x*_ ∝ ∫ ρ^4/3^(***r***)d^3^*r*. A four-dimensional connection between size and polarizability was also found based on scaling arguments related to the hard/soft acid/base theory, although it was suggested that open-shell atoms scale differently than closed shell ones^9^ due to the different role of core and valence electrons. The connection of polarizability or dispersion coefficients and volume, ionization energy, or electron affinity has also been extensively studied,^5,21−23^ since scaling of atomic properties is a key ingredient in multiple dispersion models.^24,25^ It has long been proposed^26^ that the homoatomic *C*_6_^AA^ scales as α^A^(0)^3/2^, with the combination rules for dispersion coefficients closely conforming to this relation,^27^ effectively connecting free atomic response properties to dispersion parameters. However, for fullerenes, a *C*_6_ ∝ *n*^2.75^ scaling was found (*n* being the number of carbon atoms) due to collective electrodynamical effects.^28^

In this work, the four-dimensional quantum relation between size and polarizability is generalized to many-electron atoms and molecules. This analysis will proceed as follows. First, utilizing that the finite square well (FSW) Hamiltonian can host a controlled number of bound states together with the continuum part of the spectrum, it is shown that the scaling law correctly encompasses both bound and continuum contributions. Second, this scaling law is extended to elements of the periodic table following a variational derivation, which, by construction, incorporates electron correlation effects consistently. It is shown that the four-dimensional scaling holds for free atoms and atoms in confinements, providing a fundamental justification for atoms-in-molecules (AiM) approaches. Finally, we show that a combination of the atomic four-dimensional scaling law with long-range electrostatic coupling enables us to build a constructive theory for the dipole polarizability tensor of molecular systems.

## 2 Background

The dipole polarizability is a second-rank tensor determining the dipole moment induced by an applied electric field as . For isotropic systems, this quantity reduces to a scalar α = α_*ii*_, where *i* ∈ {*x*, *y*, *z*}. For anisotropic systems, one can also define a scalar polarizability as . The straightforward calculation of the dipole polarizability of a quantum-mechanical system in its ground state |Ψ_0_⟩ with energy *E*_0_ is based on second-order perturbation theory^29^
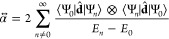
1where ⊗ indicates the dyadic vector product and the sum goes over all excited states |Ψ_*n*_⟩ with energies *E*_*n*_. The dipole operator is given by **d̂** = ∑_*j*_**d̂**_*j*_ = ∑_*j*_*q*_*j*_**r̂**_*j*_, where *q*_*j*_ and **r̂**_*j*_ are the charge and position operator of the *j*th particle, respectively.

To accurately evaluate α, one needs to take into account bound and continuum excited states, both always present for atoms and molecules. For example, the continuum contribution to the dipole polarizability of hydrogen is known to be about 20%.^30^ For many-electron cases, the calculation of polarizability is based on evaluating the change of the dipole moment with respect to an external electric field or on the ability to accurately predict excited-state wave functions with methods such as time-dependent density functional theory or linear response coupled cluster theory.^31−33^ Approaches using finite-field derivatives suffer from convergence and algorithmic issues, while excited-state calculations require excessive computational power. Therefore, calculating the dipole polarizability is still a demanding task in practice, which makes approximate methods, property correlations, and general scaling laws desirable.^34−36^

**Figure 1:**
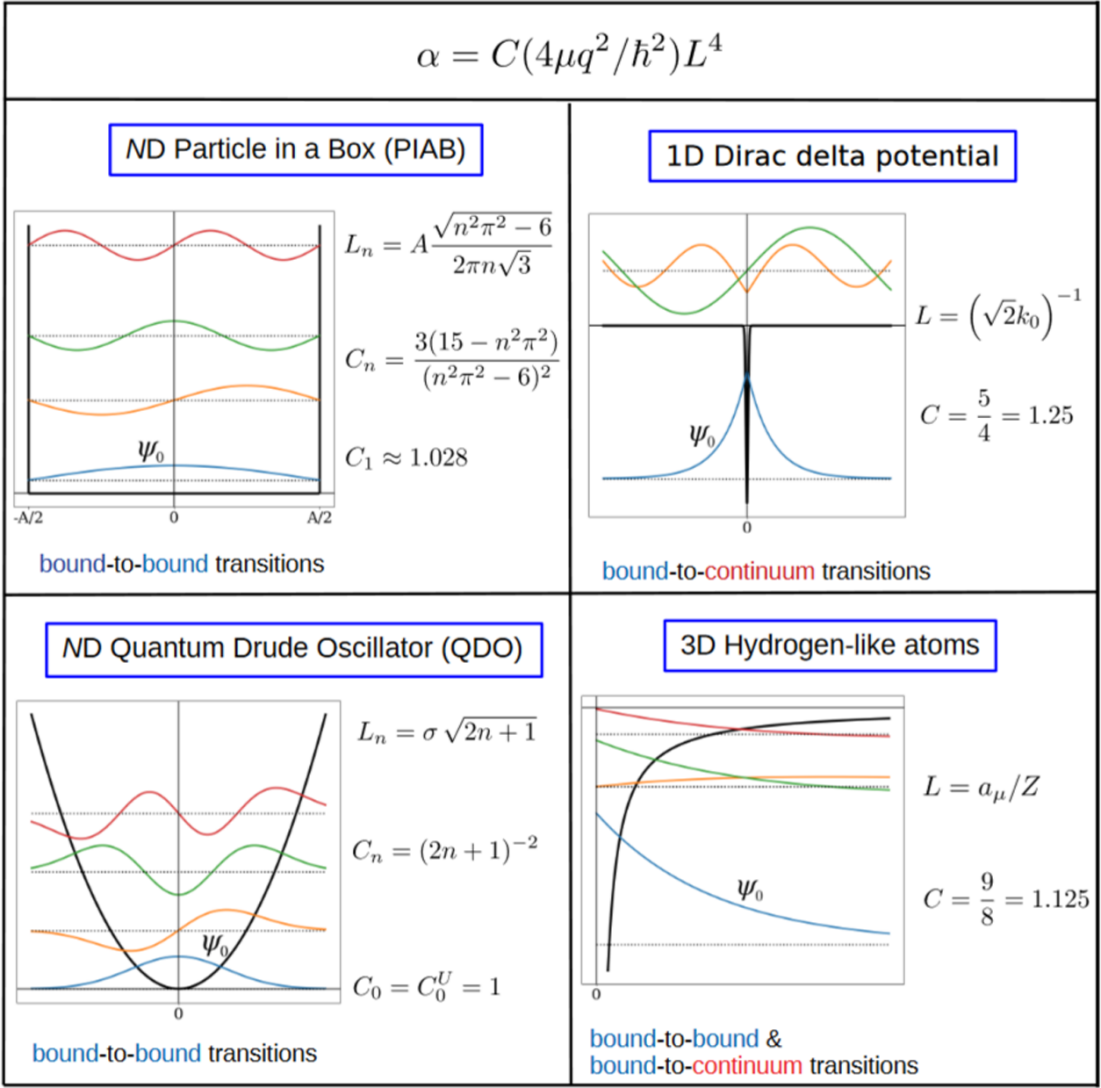
Four-dimensional scaling of dipole polarizability for the particle in a box, the Dirac delta potential, the quantum Drude oscillator and the hydrogen-like atoms. Although the spectra of these models are qualitatively different, their polarizability α is proportional to the characteristic length *L* to the fourth power, with a coefficient *C* close to unity (adapted from ref (19).).

As discussed in the Introduction, polarizability was found to be proportional to a length scale  to the fourth power for a wide range of quantum systems (Figure 1), i.e.,
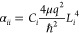
2The characteristic length is defined with the -norm of the position operator *r̂*,^19^ calculated independently for each Cartesian component *i* with respect of their individual origin *R*_*i*_, as
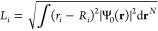
3where *N* is the system spatial dimensionality (D).

The advantage of eq 3 is that this measure is well-defined for all systems; therefore, a universal scaling law can be derived from it, encompassing model Hamiltonians as well as real atoms and molecules. However, a scaling law between size and polarizability must also describe electron correlation. For this reason, the *C*_*i*_ coefficient masks two effects: the error of representing the contributions of all transition moments by a single effective transition and the error of neglecting the correlation effects.

The validity of the effective transition approximation can be already seen by studying one-electron models: the hydrogen atom, having no electron correlation, has a *C* coefficient of 1.125, slightly deviating from *C* = 1 observed for the quantum Drude oscillator, for which a two-state model is exact. This approximation is examined in Section 3.1 on the basis of the FSW potential, a correlation-free model having a controlled ratio of bound and continuum states. The effect of electron correlation is taken into account in Section 3.2 by redefining the atomic size using a correlated operator, allowing us to study all elements of the periodic table as well as atoms in external confining potentials. Finally, a model for anisotropic molecular polarizabilities is presented, which combines the universal four-dimensional scaling law with self-consistent electrostatic screening.

## 3 Results and Discussion

### 3.1 The Interplay of Bound and Continuum Contributions in Single-Electron Models

A main challenge in obtaining response properties of quantum-mechanical systems is the full treatment of bound-to-bound and bound-to-continuum fluctuations. The four-dimensional scaling law, proposed in ref (19), overcomes this difficulty by expressing the polarizability from the ground-state electron density only. Analyzing one-electron systems with qualitatively different spectral properties, it was found that a proportionality coefficient *C* must be introduced to connect the polarizability to the fourth power of the characteristic length. While the *C* coefficient was found to be close to unity for all model Hamiltonians, a detailed analysis of this parameter as a function of continuum contributions is necessary to underline the generality of the scaling law. In this work, we present this discussion on the basis of the finite square well (FSW) potential, since this model can host an arbitrary ratio of bound and continuum excited states, making it an ideal candidate to generalize the scaling law to Hamiltonians with qualitatively different spectral properties.

The Hamiltonian of a square well of width 2*a* and depth *V*_0_ is given by
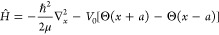
4where Θ(*x*) is the Heaviside step function. Since the potential energy is given by an even function, the eigenstates of this Hamiltonian can have either even or odd parities. Irrespective of the parameters, the model always has at least one bound state with the wave function
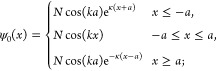
5with
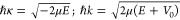
6and the corresponding normalization. Due to the continuity condition of the logarithmic derivative at ±*a*, the wave function parameters must satisfy *k* tan(*ka*) = κ. This equation is often solved by the introduction of two additional parameters , with the boundary condition taking the form
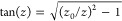
7

Equation 7 can have multiple solutions depending on the value of *z*_0_, with the *z* corresponding to the ground state taking a value in the interval [0; π/2]. The number of bound states is given by , with  representing the floor function.

For the purpose of this analysis, only the polarizability in the ground state is considered, which means that a single parameter  is sufficient to characterize the state of the system.^37^ If *z*_0_ → 0, the potential well is shallow and hosts only one bound state. Accordingly, all transitions are bound-to-continuum. In the opposite limit, e.g., *z*_0_ → ∞, the model reduces to the particle-in-a-box problem, having an infinite number of bound and no continuum states. Varying *z*_0_, therefore, enables us to unify the discussion of one-electron model systems with regard to the effect of continuum states on the scaling law of dipole polarizability.

**Figure 2:**
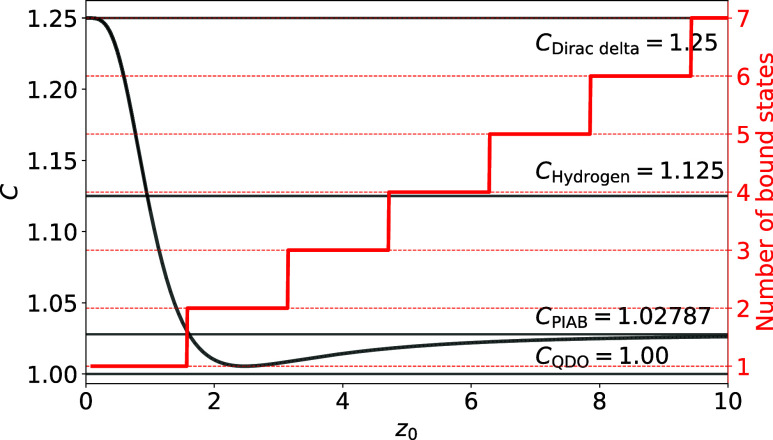
*C* coefficient (left axis) and the number of bound states (right axis) of a finite square well potential as a function of the parameter *z*_0_. The *C* value of the particle-in-a-box (PIAB), the Dirac delta potential, the quantum Drude oscillator (QDO) and the hydrogen atom is also shown.

Consequently, to describe the ground state of the FSW, one first needs to obtain the lowest value of *z* from eq 7 for a given value of *z*_0_. Having done this, the ground-state wave function can be constructed and *L*^2^ is obtained by directly evaluating ⟨Ψ_0_|*x̂*^2^|Ψ_0_⟩. The polarizability of the ground state of the FSW was obtained analytically by Maize et al.^38^ Due to the length of the equation, we do not reproduce it in this paper. However, the polarizability of the groud state can be obtained exactly from it using the values of *z* and *z*_0_. Finally, the *C* coefficient is obtained from the ratio of polarizability and *L*^4^ as prescribed by eq 2. This process was repeated for a set of *z*_0_ values between 0 and 10, the resulting *C* coefficients, together with the corresponding number of bound states, are shown in Figure 2.

Two interesting conclusions can be drawn from Figure 2. First, the values of *C* do not deviate significantly from unity for any value of *z*_0_, with the maximal value of *C* = 1.25 reached in the limit *z*_0_ → 0 (equivalent to a Dirac delta potential; see Appendix C for the derivation of the limit). The deviation from unity is determined by the qualitative properties of the spectrum of the Hamiltonian, with *C* = 1 for the harmonic oscillator and *C* being slightly larger for systems also possessing continuum excited states–the range of possible *C* values in Figure 2 is in line with this hypothesis. Second, the value of the *C* coefficient quickly converges to the limiting value dictated by the particle-in-a-box model. Notably, the scaling properties of a potential hosting only four bound states are practically identical to the one valid for the infinite bound states of the particle-in-a-box.

By controlling the number of bound states, the FSW model serves as a unifying model for one-electron Hamiltonians, allowing the study of the effect of bound and continuum part of the spectrum on the polarizability scaling systematically. The values of *C* do not deviate significantly from unity for any value of the model parameters, confirming the robustness of the four-dimensional scaling law for one-electron models. However, a general formalism for atoms and molecules needs to account for electron correlation effects not present in any single-electron model. In the next section, the effect of electron correlation will be incorporated into the definition of size, allowing the extension of the four-dimensional scaling for all elements in the periodic table.

### 3.2 Correlated Scaling Law for Many-Electron Atoms

**Figure 3:**
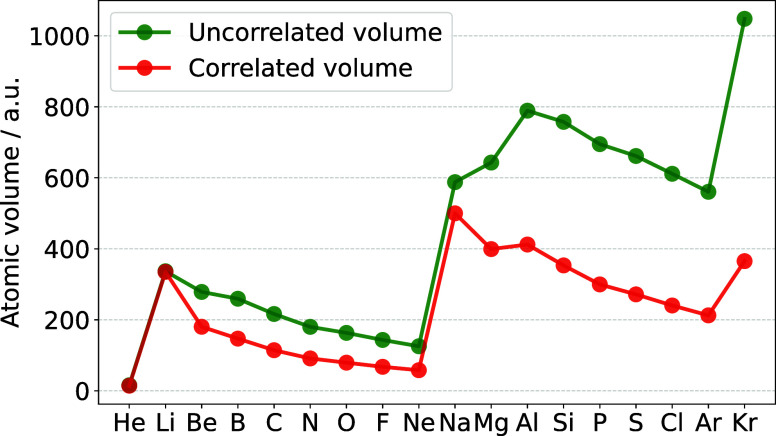
Comparing the atomic volumes (defined as *V* = 4π(*r*^2^)^3/2^/3) obtained from correlated and uncorrelated size descriptors. The effect of electron correlation, described with the 2RDM expectation value in eq 10, is increasing with the principal quantum number.

In Section 3.1 it was shown that a unified discussion of single-particle model systems can be achieved by studying the finite well potential. However, to extend the formulation to many-electron atoms, we must consider that eq 2, in its original proposed form, defines the system size via a one-electron operator in eq 3. The need to include contributions from two-electron terms was already noted by Lekkerkerker et al.,^39^ who proposed an alternative definition of size from the ground-state wave function Ψ. Analogously to *L* in eq 3, an atomic size *R* accounting for electron correlation can be defined as
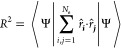
8

, with *N*_e_ representing the number of electrons in the system. Notably, the correlation contribution to the size, i.e., the difference between the sizes obtained from eqs 3 and 8 due to the inclusion of the cross-terms *x*_*i*_*x*_*j*_ was already pointed out by Vinti^40^ and was numerically shown to be significant for heavier noble gases.^39^ Indeed, having more electrons leads to a greater correlation contribution, as seen by the general trend of a growing difference between correlated and uncorrelated volumes with increasing atomic number in Figure 3. This is in agreement with the empirical finding in ref (19), where the *C* coefficient connecting size and polarizability was found to be proportional to the principal quantum number.

**Figure 4:**
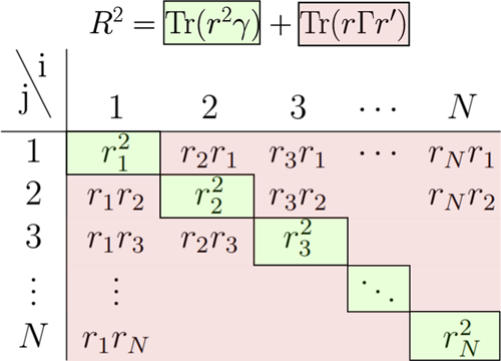
Contribution of one-electron and two-electron terms to the *R*^2^ expectation value.

With the correlated definition, the atomic dipole polarizability is still proportional to the atomic size (normalized by the number of electrons *N*_e_)
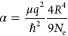
9

The derivation of eq 9 is discussed in Appendix A based on ref (39). For one-electron systems, eq 9 is equivalent to (2), with a factor of 9 in the denominator appearing due to eq 9 assuming a spherical symmetry in the Cartesian components.

The numerical implementation of eq 9 is based on the fact that the expectation value can be reformulated in terms of the (spin-traced) one- and two-electron reduced density matrices^41^ (1RDM γ and 2RDM Γ) as (Figure 4)
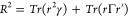
10with the first, 1RDM-dependent term accounting for the uncorrelated *r*^2^ size and the 2RDM-dependent term introducing the cross-terms. We have obtained the unrelaxed reduced density matrices projected onto the atomic orbital basis on the coupled cluster singles and doubles (CCSD) level using the implementation in PySCF,^42−44^ with the aug-cc-pVTZ^45,46^ Gaussian atomic basic set. We note here that the N-representability of general density matrices is an open question in the electronic structure theory community.^47^ Specifically, 2RDMs obtained from coupled cluster approaches with response formulation can be unphysical.^48^ To ensure the correctness of our results, we have instead obtained the 2RDM from a direct projection, and ensured that the trace condition is fulfilled for all calculations. Moreover, the observation that our numerical results are in good agreement with the ones of Lekkerker et al.^39^ and Cambi et al.^49^ further ensures the reliability of our results.

A direct application of eq 10 with the total number of electrons does not yield satisfactory results, with most atomic polarizabilities predicted only within a factor of 2. Notably, polarizabilities are underpredicted, with the magnitude of the error increasing as the principal quantum number increases. This observation is consistent with the physical picture that polarizability is almost exclusively determined by the dipole–dipole fluctuations of the valence shell, with the contributions from the core electrons being often negligible. To account for this effect, an effective number of electrons *N*_e_^eff^ can be defined from eq 9 by setting it to give the exact polarizability (as opposed to defining the polarizability via the effective number of electrons). The effective number of electrons defined this way closely resembles the number of valence electrons, as seen in Table 1. The difference between the valence and the effective electron numbers can be attributed either to the breakdown of the variational ansatz or to the effect of core electrons on the valence shell. In general, the difference between the valence and effective electrons is larger for the first-row elements than for the second-row ones, with the response in the first row corresponding to a smaller number of effective electrons.

**Table 1:** Effective Number of Electrons *N*_e_^eff^ Resembles the Number of Valence Electrons *N*_e_^val.^ for Elements in the Periodic Table

element	*N*_e_^eff^	*N*_e_^val.^	element	*N*_e_^eff^	*N*_e_^val.^	element	*N*_e_^eff^	*N*_e_^val.^
He	1.74	2	Li	0.92	1	Be	1.76	2
B	2.46	3	C	3.20	4	N	3.61	5
O	4.17	6	F	4.77	7	Ne	5.47	8
Na	1.58	1	Mg	2.69	2	Al	3.43	3
Si	4.34	4	P	5.22	5	S	5.89	6
Cl	6.63	7	Ar	7.40	8	Kr	10.0	8

While the effective number of electrons is introduced here in an *ad hoc* way, the necessity for this correction can be rationalized, for example, by noting that the numerator in eq 9, i.e., the atomic size, is largely determined by the valence shells, whereas their relative contribution to the total number of electrons in the denominator is diminishing with increasing the principal quantum number. The effective number of electrons in Table 1 follows the same periodic trend as the number of valence electrons, thereby ensuring a consistent scaling of the numerator and the denominator for all elements in the periodic table.

Naturally, the use of the *N*_e_^eff^ values in Table 1 in eq 9 would lead to an exact prediction of polarizability by construction. Nevertheless, our approach can be confirmed by noting that the effective number of electrons obtained by our approach are within 15% of those of Cambi et al.,^49^ defined using the combination rules of dispersion coefficients. Remarkably, while the effective number of electrons was obtained from the dispersion coefficients in ref (49), the scheme proposed here relies only on atomic size and polarizability, allowing the use of more accurate electronic structure methods.

**Figure 5:**
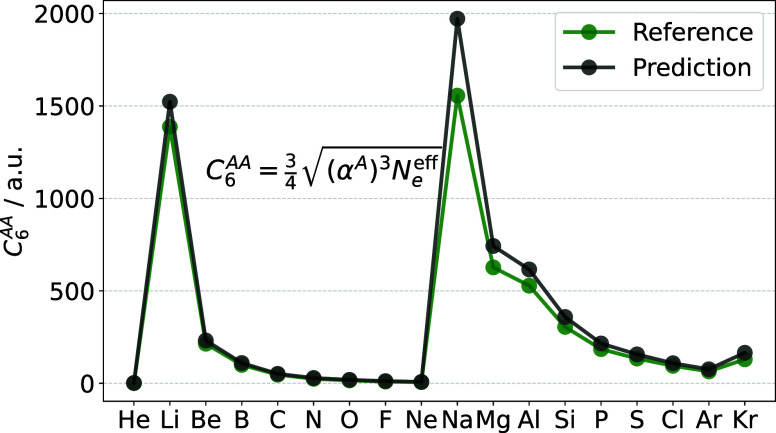
Comparing the prediction of the Slater–Kirkwood formula with the effective number of electrons for the homoatomic dipole–dipole dispersion coefficient with reference data.

The concept of the effective number of electrons can be directly applied in the Slater–Kirkwood formula^49,50^ to construct interatomic parameters from polarizability. The general combination rule for the multipole dispersion coefficients of orders  between two atoms (with ℏ = *m* = 1 for atomic response) is given as
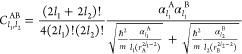
11As was already pointed out in ref (39), this equation can be obtained by regarding eq 9 as the result of an Unsöld approximation with the average excitation energy  and using the Casimir-Polder formula. For the homoatomic dipole–dipole dispersion coefficient *C*_6_^AA^ (), using the effective number of electrons as before, this reduces to the simple expression–in atomic units −
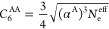
12Conversely, eq 9 provides a direct way to obtain the effective number of electrons *ab initio*. Figure 5 shows that eq 12, with the number of effective electrons of Table 1 obtained from the connection between polarizability and size, gives a good prediction for atomic dipole–dipole dispersion coefficients.

In conclusion, it is established that the dipole polarizability of free atoms scales with the fourth power of the correlated atomic size and is inversely proportional to the effective number of electrons participating in the dipolar response. Dispersion coefficients can also be computed by using polarizability and effective number of electrons, without the need for time-dependent calculations. This provides yet another successful example of utilizing the insight gained from quantum-mechanical scaling laws to establish new models from simpler properties.

### 3.3 Scaling of Polarizability for Confined Atoms

A major application of the four-dimensional scaling is the definition of atomic polarizabilities within atoms-in-molecules (AiM) approaches. For example, the exchange-dipole model (XDM^24^) and the Tkatchenko-Scheffler (TS)^25^ dispersion schemes both rely on accurate atomic polarizabilities. Within the TS approach, the dispersion energy of a system is expressed as the sum of pairwise damped interatomic dipole–dipole interactions
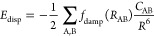
13with the *C* coefficients obtained from integrating over the imaginary frequencies of the atomic dipole polarizabilities approximated by a quantum Drude oscillator model as
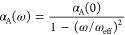
14Due to the analytical properties of the quantum Drude oscillator model, the effective frequency ω_eff_ can be obtained from α(0) and *C*_6_. The problem of determining the dispersion interaction energy then reduces to the problem of defining α(0) and *C*_6_ for each atom in a molecule, with the scaling of *C*_6_, in turn, defined with respect to the scaling of α(0) by the Casimir-Polder integral.^51^ These considerations lead to the scaling of the dipole polarizability being the only free choice in the model. The authors of the TS model proposed a scaling law based on the classical connection between dipole polarizability and size, i.e.,
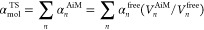
15where the sum runs over all atoms in the molecule. The weights (*V*_*n*_^eff^/*V*_*n*_^free^) measuring the volume ratio of the atom in a molecule to the free atom in vacuum are most often obtained by Hirshfeld partitioning of the electron density,^52^ but other partitioning schemes as well as machine learning approaches have also been presented.^53,54^ Based on the four-dimensional scaling law, it was already shown in ref (19). that a simple rescaling of the volumes yields better predictions for molecular polarizability
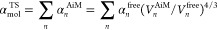
16

The study of atoms in confinement provides a way for a more rigorous analysis of the scaling of polarizability of atoms in molecules, since the addition of a confining potential to the free atomic Hamiltonian can be used to model the effect of chemical bonding.^55^ For this reason, the response properties, especially the polarizability, of atoms and molecules in confinement have been the focus of some recent studies.^56−59^

To investigate the scaling law between size and polarizability in confinement, eq 8 was evaluated along an increasing confinement radius, with the polarizability being obtained by calculating the response to an additional external perturbing electric field at each step. The form of the confining potential is given in eq 17, using a soft wall that ensures that the long-range limit of the potential energy does not qualitatively differ from free atoms.^59^
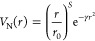
17

**Figure 6:**
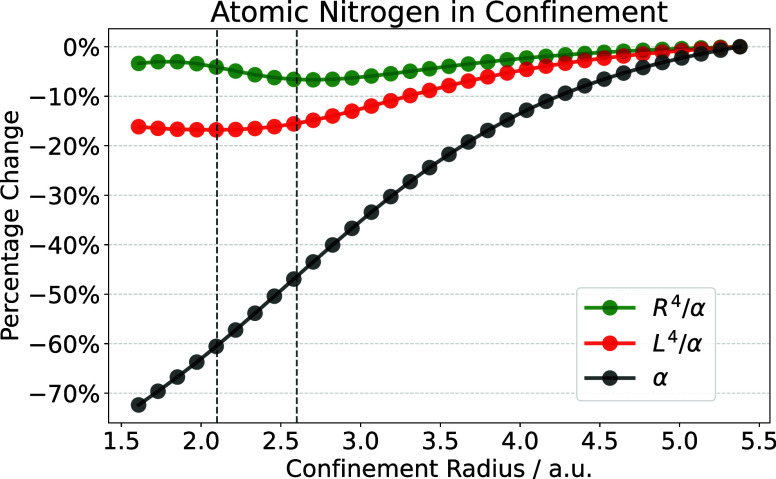
Percentage change in polarizability as well as the two possible invariants as a function of the confinement radius for atomic nitrogen, calculated on the UCCSD/aug-cc-pVTZ level. The range where the confinement radius is most relevant for molecular application is marked in by dashed lines.

Figure 6 shows the polarizability as well as the ratio of polarizability and *R*^4^ and *L*^4^ for the free (quartet) nitrogen atom as a function of the confining radius *r*_0_. The ratio between the different four-dimensional sizes and the polarizability would be constant if eq 9 (or eq 2) were to hold exactly. Therefore, deviations from a flat line measure the error of the scaling law. The stiffness parameter was chosen as *S* = 4 and the softness parameter was set as γ = 0.1, with the results independent of the choice of these hyperparameters (see Appendix B). The calculations were performed on the UCCSD/aug-cc-pVTZ level using PySCF.^42,43^ Polarizability was approximated using a single-point derivative with a field strength of *E* = 0.01 au. These parameters were chosen so that not only a wide range of the confinement is studied but also commonly encountered AiM volumes are present, as discussed in the Appendix.

It can be concluded from Figure 6 that, while the *R*^4^/α curve is not constant, the error introduced by eq 9 is approximately 7% in the full range and less than 3% in the confinement range most significant for AiM models. Compared to the almost 4-fold increase in polarizability values, this shows that eq 9 maintains good accuracy along the confinement. Interestingly, the negative slope part of the curve closely corresponds to the chemically relevant part of the confinement radius. Nevertheless, the 3% error introduced by the scaling law in this range is comparable in magnitude to the accuracy of polarizabilities obtained by density functional theories.^60^ Therefore, defining the polarizability in AiM models based on eq 9 introduces a relatively small error for practical computational models.

Combining the scaling law of polarizability with the Slater–Kirkwood formula for the dipole–dipole dispersion coefficient in eq 11, with the assumption that the number of effective electrons remains constant, leads to the appropriate scaling law for the *C*_6_ coefficient
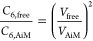
18Notably, this is consistent with both the original approximation in the Tkatchenko-Scheffler model^25^ (obtained with the Casimir-Polder integral and assuming a polarizability scaling proportional to volume) as well as the numerical results of Gould^5^ who found that the difference of the power law in polarizability *p*′ and the *C*_6_ coefficient *p* are connected as *p*′ = *p* – 0.615, with our derivation conforming to . The small mismatch in the exponents can be attributed to the contribution of higher-order variational terms or the change in the effective number of electrons on our end, or to the simpler definition of atomic volume and the lack of the soft decay of the confining potential in ref (5).

Finally, it should be noted that the invariant constructed from the uncorrelated size metric, e.g., *L*^4^/α, shows a more significant deviation as a function of the confinement radius, highlighting the need for correlation contributions. However, while partitioning electron densities is routinely available in electronic structure codes, partitioning the 2RDM is not generally performed. Moreover, the 2RDM is only defined for correlated electronic structure methods, whereas mean-field calculations (including most density functionals) yield only electron densities. As can be seen from the behavior of the two invariants in Figure 6, defining the AiM polarizabilities from the electron density is a reasonable compromise. The accuracy of this approximation, coupled with long-range electrostatic screening, will be explored in the next Section.

### 3.4 Molecular Polarizability from Screened Atomic Response

The preceding results allow for the combination of the correct scaling properties of local polarizability with a model for long-range interactions. The quantum-mechanical four-dimensional scaling law was shown to be valid for one-electron model systems and atoms in confinement in this work. It was also concluded in Section 3.3 that neglecting correlation contributions to the AiM scaling is a reasonable practical approximation. This provides a rigorous justification for defining AiM polarizability using free atomic values rescaled with four-dimensional “sizes”, as was also suggested in some previous works.^19,61,62^

In principle, molecular polarizability should be described using two-point response functions accounting for the nonlocal nature of the electronic response.^63^ While powerful and in the focus of different groups recently,^64,65^ this theory is quite elaborate, and the full nonlocal polarizability is only known for the homogeneous electron gas.^12^ Practical models therefore rely on various levels of coarse-graining. Of these, assigning polarizabilities to atomic centers is the oldest and the most widely used approach.^35,66^ This, however, suffers from two shortcomings, namely, the ambiguity of defining atomic polarizabilities and the fact that long-range delocalization and charge transfer effects are not directly amenable to atomic coarse-graining. These problems have been addressed either by including bond-dependent contributions,^67^ or by evaluating charge flow effects.^68,69^

Overall, modeling polarizability with a sum of adequately scaled interacting AiM polarizabilities is a useful model, and the accuracy of this approach is evaluated in this section. To this end, we have used the equilibrium geometries from the QM7–X database.^70^ This data set, which contains 4.2 million organic structures with no more than seven heavy (C, N, O, S, Cl) atoms, was designed to describe the variation of 42 physicochemical descriptors over a significant subset of the chemical compound space, accounting for the differences between isomers and the effect of out-of-equilibrium geometry distortion. For the purpose of the present work, only the 42 thousand equilibrium geometries were used. The AiM volumes were obtained using the PBE functional, while the overall molecular polarizability was calculated by us using density functional perturbation theory (DFPT) with the PBE0 functional. The density functional theory calculations and the density partitioning were done using the FHI-aims software.^71,72^ The self-consistent screening (outlined below) was performed using a modified version of libMBD,^73^ a portable library that implements the many-body dispersion correction scheme. The predictive power of AiM approaches also depends on the choice of the partitioning scheme. In particular, the Hirshfeld partitioning has a degree of arbitrariness in constructing the promolecular density, therefore, the iterative variant should be used for systems with nonzero net charge or large polarization effects,^52,74,75^ and this is the scheme that was used in this work.

**Figure 7:**
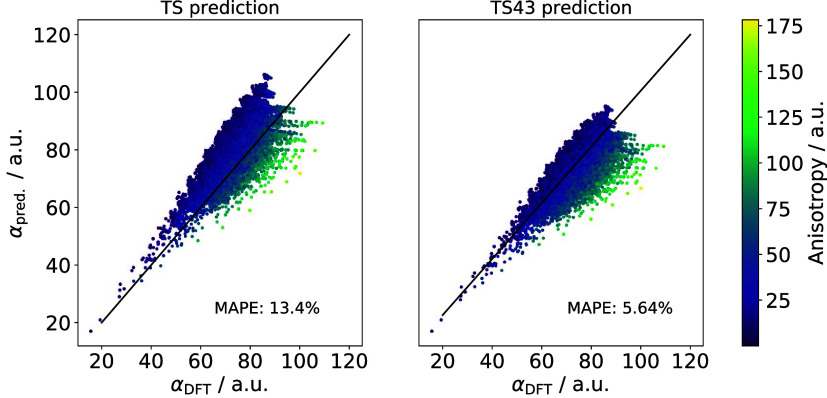
Comparing the trace of dipole polarizabilities predicted by the “TS” and “TS43” methods versus PBE0 reference values on the QM7–X data set of small organic molecules.^70^ The color of each point is determined by the anisotropy of the polarizability tensor. The mean absolute prediction error (MAPE) for each method is also shown.

The simplest model, based on the unmodified Tkatchenko-Scheffler (TS) approximation, expresses the molecular polarizability as a sum of rescaled atomic values, the rescaling being of the form of the originally proposed three-dimensional proportionality (cfi. eq 15). Based on the validity of the four-dimensional scaling law for atoms in confined potentials as well as previously published results,^19^ it is expected that rescaling the volume ratios as prescribed by eq 16 would improve the molecular polarizability prediction. In fact, a significant improvement can be obtained from the unmodified “TS” to the rescaled “TS43” scheme, as seen in Figure 7.

Although a mean absolute prediction error (MAPE) below 6% is comparable to the errors of many density functionals,^60^ a simple sum of atomic polarizabilities cannot capture long-range electrostatic interactions. This is exemplified in Figure 7 by the color of each point corresponding to the anisotropy of the (static) polarizability tensor Δ**α** as obtained from the DFPT calculation as
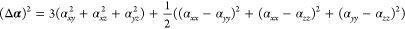


Treating the interaction between local atomic polarizabilities using a self-consistent dipole–dipole screening model allows for the inclusion of anisotropic effects. Within this approach, the polarizability of atom *j* is obtained using a Dyson-like screening, with contributions from all other atoms *k* ≠ *j*
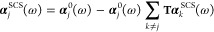
19The dipole–dipole interaction tensor *T* is obtained from considering a Gaussian charge distribution on each atom, in order to avoid possible divergences due to overlapping polarizabilities
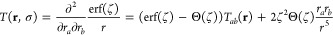
20where ***r*** is the distance vector connecting the two atoms, and the σ “interaction” width is obtained from the individual Gaussian widths as
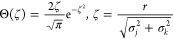
21The individual Gaussian σ parameters were obtained, following the prescription of Mayer, from AiM polarizabilities^76^
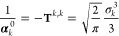
22

Combining the long-range self-consistent screening with the four-dimensional scaling of local atomic polarizabilities, we obtain a method we called “SCS43”, which we analyze in the following.

**Figure 8:**
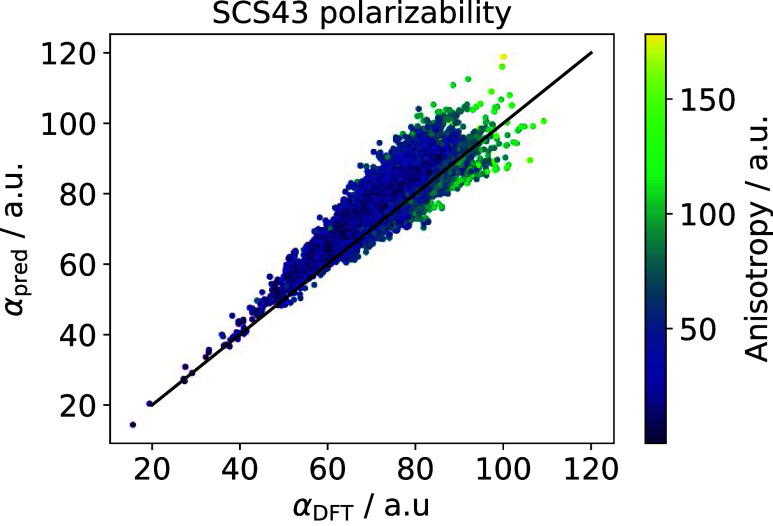
Trace of dipole polarizability tensor predicted by the “SCS43” methods versus PBE0 reference values on the QM7–X data set of small organic molecules.^70^ The color of each point shows the anisotropy of the polarizability tensor.

From Figure 8 one can conclude that an accurate prediction of molecular polarizability can be achieved if both the four-dimensional scaling is respected and the long-range screening effects are taken into account. Although an improvement in the prediction of molecular polarizability due to the four-dimensional scaling law was already presented in ref (19), the large data set in this work enables us to quantify the effects of the anisotropy of the response. In particular, the prediction errors of the “TS43” method correlate with the anisotropy of polarizability, having a correlation coefficient of *R*^2^ = 0.78, while the self-consistent screening in “SCS43” eliminates this systematic error, having a *R*^2^ value of 0.11, confirming that a local model for molecular polarizability can not account for largely anisotropic structures, but the introduction of self-consistent screening dispenses with this error. The molecules that are worst predicted with the “SCS43” approach all have large and highly anisotropic polarizability tensors. These structures also contain multiple double and triple bonds. This is in line with expectations that charge-transfer terms are needed to treat extended conjugated structures, as was done in previous works.^76^ However, our “SCS43” approach can predict polarizabilities in the chemical compound space of small organic molecules with reasonable accuracy.

In conclusion, a combination of the four-dimensional scaling of AiM polarizabilities with a self-consistent screening approach gives a predictive model for molecular polarizabilities requiring solely the ground-state electron density. The predictive power of the presented framework could still be improved by tuning the atomic parametrization and the interaction potential together, including long-range charge transfer effects. This could be examined in future work.

## 4 Conclusions

A constructive model based on a combination of local polarizabilities with long-range interactions is essential for applications involving large, complex molecular systems. Such a model relies on a physically correct scaling law of atomic polarizabilities as well as an accurate account of their long-range coupling. In this work, both of these components were studied.

Using the finite square well potential as a unifying model for one-electron systems, we showed that the four-dimensional scaling law between polarizability and system size is largely independent of the nature of dipole fluctuations that determine the polarizability.

Extending the derivation to many-electron atoms, the role of electron correlation was discussed. In particular, accounting for two-electron contributions was found to be crucial for atoms having many electrons in the valence shell. The Slater–Kirkwood formula for dipole–dipole dispersion was also revisited, showing good agreement with reference results if an effective number of electrons is considered. The connection between size and polarizability also holds for atoms in soft-wall confining potentials, further highlighting the generality of the four-dimensional scaling law and providing a theoretical justification for atoms-in-molecules approaches.

Finally, it was shown that a good prediction of the total molecular polarizability can be achieved with a method that locally respects the four-dimensional scaling law and accounts for long-range effects using dipole–dipole screening. In particular, this approach was shown to achieve an 8% prediction accuracy for organic molecules without an observable systematic error as a function of the total polarizability anisotropy.

In conclusion, we have presented multiple theoretical and computational advances toward understanding the scaling of dipole polarizability with quantum-mechanical size:

- A full solution of the particle-in-a-box model system is presented as a unifying model for one-electron Hamiltonians having controllable bound and continuum contributions to the polarizability;
- A variational derivation is revisited for many-electron atoms, providing a link between atomic size, electron correlation effects, effective number of electrons, and polarizability, all while following the four-dimensional scaling;
- The scaling is proven to hold for atoms in soft-wall confining potentials, providing an attractive physical model for the concept of atoms-in-molecules; and finally
- The results obtained from free atoms and atoms in confinements are combined with long-range electrostatic screening to give a predictive model for the anisotropic dipole polarizability of organic molecules.

These findings constitute an overarching framework for the theory and application of the four-dimensional size scaling of the dipole polarizability for model systems, atoms, atoms in molecules, and molecules.

## Supporting Information Available

Derivation of the scaling law; assessing choices of calculation settings; derivation of the *z*_0_ → 0 scaling limit of the FSW model, the two worst predicted molecules with the “SCS43” approach (PDF)
