# Supplementary material for: Four-Dimensional Scaling of Dipole Polarizability: From Single-Particle Models to Atoms and Molecules
Source: J Chem Theory Comput. 2024 Jul 17;20(15):6621–31. doi: 10.1021/acs.jctc.4c00582 (PMC11325554; doi:10.1021/acs.jctc.4c00582)
Supplement: Supplementary file 1 — ct4c00582_si_001.pdf [file ct4c00582_si_001.pdf]

Four-Dimensional Scaling of Dipole Polarizability: From Model Systems to Atoms and Molecules

–Supporting Information–

Szabolcs Góger[a], Mohammad Reza Karimpour[a], Alexandre Tkatchenko[a,*]

[a] Physics and Materials Science Research Unit, University of Luxembourg,

L-1511 Luxembourg, E-Mail: alexandre.tkatchenko@uni.lu

* Corresponding author

Date: July 9, 2024

Appendix A.

Eq. (9) can be reached by following two different approaches: using a perturbative argument with average excitation energies or by following a variational approach. The perturbative solution was first proposed by Vinti,[40] and was applied to the extensive study of model systems to generalize the four-dimensional scaling law.[19] In this Appendix, the variational derivation of Lekkerkerker[39] is summarized.

The static 2^ℓ polarizability gives the connection of the induced moment $Q_{\ell,m}$ to the strength $A_{\ell,m}$ of an external perturbing potential

$$\phi(\boldsymbol{r}) = \sum_{\ell,m} A_{\ell,m} r^\ell Y^*_{\ell,m}(\theta,\phi) \ . \tag{23}$$

In general, polarizability also depends on m, but for spherically symmetric systems, the label ℓ is sufficient. The response of the quantum mechanical system can be studied in terms of the perturbing operator $\hat{V}_\ell = -\sum_{i=1}^{N_e} r_i^\ell Y^*_{\ell,0}(\theta)$. Within first order perturbation theory, one can obtain the polarizability from the first order correction to the wavefunction χ_ℓ using the corresponding multipole moment operator as

$$\alpha_\ell = -2\langle \Psi_0 | \hat{Q}_{\ell,0} | \chi_\ell \rangle \ . \tag{24}$$

χ_ℓ can be obtained, for example, by solving the differential equation connecting it to the perturbing operator

$$(\hat{H}_0 - E_0)\chi_\ell + \hat{V}_\ell \Psi_0 = 0 \ . \tag{25}$$

This direct solution is called the Dalgarno-Lewis technique, and it is able to provide solutions for a wide range of model systems . For the purpose of the current derivation, Eq. (24) is considered as the stationary condition of the functional

$$L[\chi_\ell] = \langle \chi_\ell | \hat{H}_0 - E_0 | \chi_\ell \rangle + \langle \chi_\ell | \hat{V}_\ell | \Psi_0 \rangle + \langle \Psi_0 | \hat{V}_\ell | \chi_\ell \rangle \ . \tag{26}$$

Lekkerkerker proposed a linear ansatz for the first order correction, *e.g.*, $\chi_\ell = \lambda_\ell V_\ell \Psi_0$, resulting in the stationary condition

$$\lambda = -\frac{\langle \Psi_0 | \hat{V}_\ell^2 | \Psi_0 \rangle}{\langle \Psi_0 | \hat{V}_\ell (\hat{H}_0 - E_0) \hat{V}_\ell | \Psi_0 \rangle} \, . \tag{27}$$

Substituting Eq. (27), together with the linear ansatz, into Eq. (24), gives (after some tedious operator algebra, for which we refer directly to Ref. 39) the final result

$$\alpha_\ell = \frac{4 (R^{2\ell})^2}{(2\ell + 1)^2 \ell (r^{2\ell - 2})} \, . \tag{28}$$

Eq. (9) is the $\ell = 1$ case of Eq. (28).

Appendix B.

The effect of the confinement potential on polarizability and atomic sizes was presented in the main text for the case of a nitrogen atom with a specific set of confinement parameters. The effect of the confinement potential on polarizability was discussed in depth in Ref. 59. In our testing of the hyperparameters, we have found good agreement with the results presented in that work (see Fig. S2 for the effect of the stiffness of the potential), with our conclusions of the scaling law being largely independent of the actual parameters chosen.

The choice of the nitrogen atom for demonstration purposes was motivated by two reasons. First, nitrogen is a common element in both organic and inorganic structures, so the properties of nitrogen under confinement are representative for realistic molecules. Second, the ground state of the nitrogen atom can be described with only a single electronic microstate, ensuring that the coupled cluster method is fully adequate because of the relative unimportance of static correlation. In contrast, the ground-state electronic configuration of carbon is known to change during confinement,[59] necessitating more elaborate electronic structure methods. Nevertheless, the observation that the correlated invariant describes the

response along confinement better than the uncorrelated one can be observed for other elements, even if not directly relevant to chemical bonding phenomena. Fig. S1 shows this for the case of argon ($S = 4.0, \gamma = 0.1$)

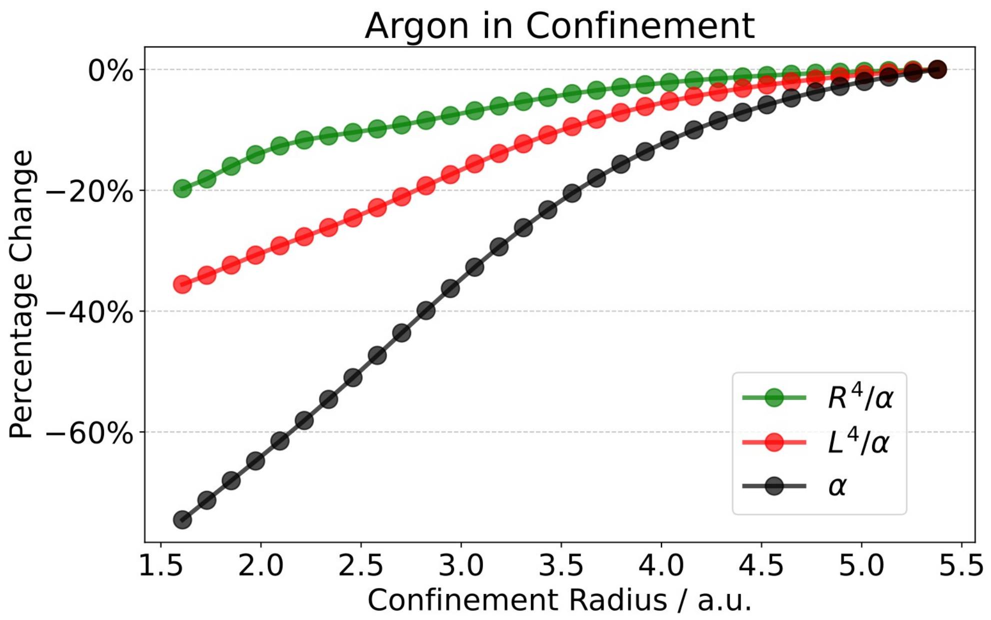

Figure S1: The change of polarizability as well as the two invariants for an argon atom in confinement.

Finally, to establish chemically relevant ranges of the confinement radius, we have analyzed the distribution of Hirshfeld volumes in the QM7–X dataset, describing a subset of the chemical compound space of organic molecules having no more than seven heavy (O, C, Cl, S, F, N) atoms. The dataset contains about 42 k equilibrium structures, which were used in this work. The Hirshfeld volumes, calculated using the expectation value of the operator $\hat{\boldsymbol{r}}^3$ were taken from the original reference.

It should be noted that the numbers in Fig. S3 are not directly comparable to the expectation values obtained in the confinement calculation, partly due to a different quantum chemical level and partly due to the fact that we have calculated the expectation values of the operator $\hat{\boldsymbol{r}}^2$, and the Hirshfeld volumes were approximated simply as $V \approx (\hat{\boldsymbol{r}}^2)^{3/2}$. However, this approximation was found to be quite accurate for small organic molecules in Ref. 19.

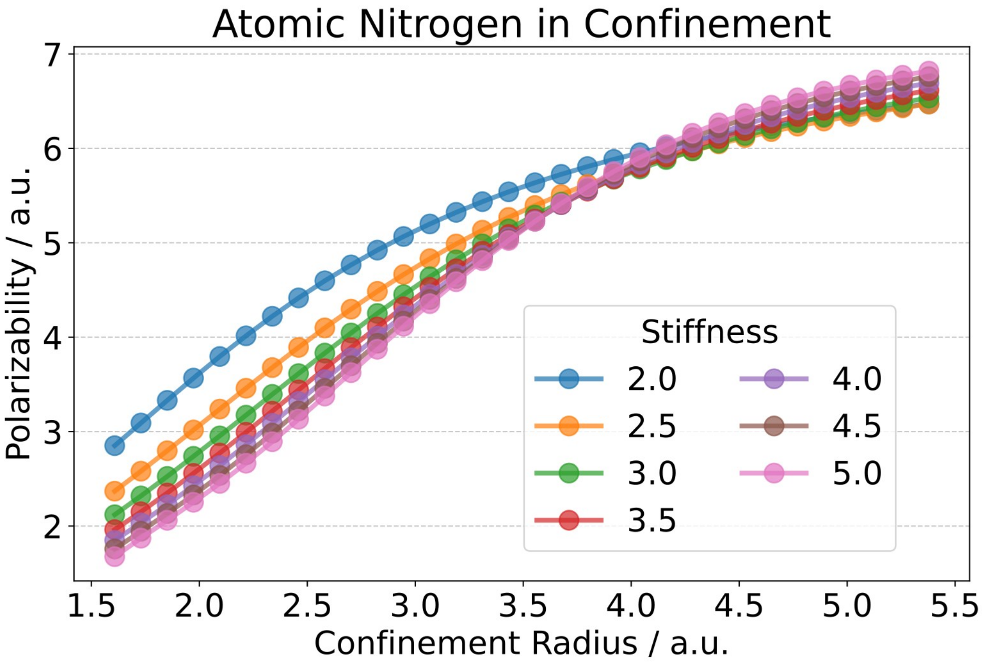

Figure S2: The effect of the stiffness of the confining potential on the polarizability of nitrogen.

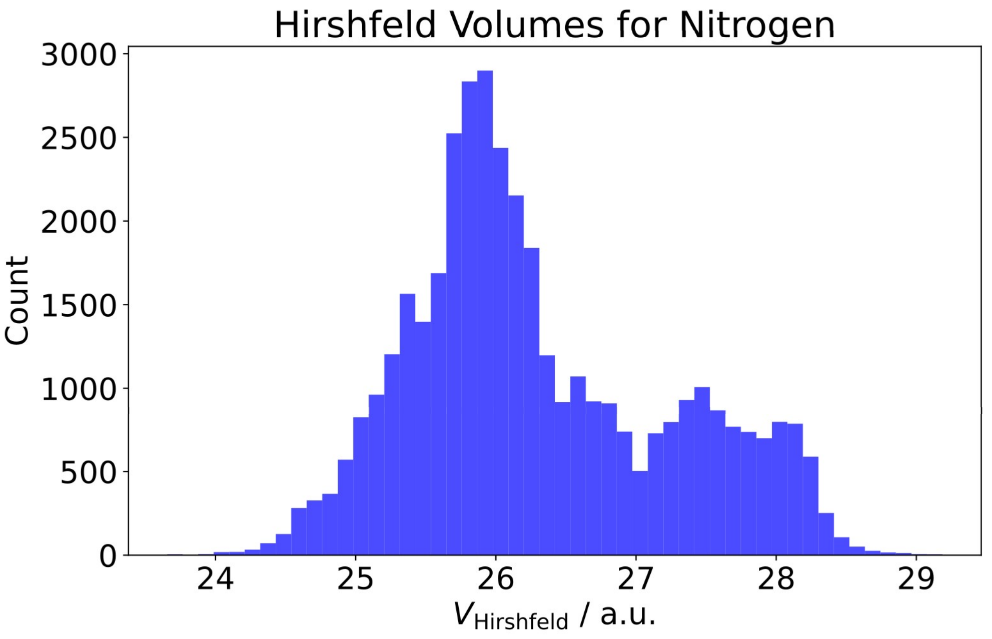

Figure S3: The distribution of the Hirshfeld volumes of nitrogen atoms in the QM7-X dataset, representing a subset of the chemical compound space of small organic molecules with up to 9 heavy atoms.

In general, the region representing the chemically important region of polarizability in Fig. 6 was set at $r_0 \in [2.1, 2.6]$ a.u., roughly corresponding to the Hirshfeld volume range of $[24, 29]$ a.u., encompassing essentially all the points in Fig. S3.

Appendix C.

The transcendental equation (7) for the energy eigenvalues of even states of a particle in a finite square well can be written in the simpler form

$$z^2 \tan^2(z) = z_0^2 - z^2 \quad \Rightarrow \quad z^2[1 + \tan^2(z)] = z_0^2 \quad \Rightarrow \quad z = z_0 \cos(z) \ , \tag{29}$$

considering that $z, z_0 > 0$. For small values of z_0 and z, the function $z/\cos(z)$ can be approximated with $z/(1 - z^2/2)$ (see Fig. S4). Thus, Eq. (29) becomes

$$z = z_0 \left(1 - \frac{z^2}{2}\right) \ , \tag{30}$$

with the simple solution

$$z = \frac{1}{z_0}\left(\sqrt{1 + 2z_0} - 1\right) \ . \tag{31}$$

Having the solutions for z and relations

$$k = \frac{1}{az_0}\left(\sqrt{1 + 2z_0} - 1\right) \quad , \quad \kappa = \sqrt{\left(\frac{z_0}{a}\right)^2 - k^2} \ , \tag{32}$$

one can obtain the lengths scale L and polarizability α and replace them into the relation

$$C(k, \kappa) = \frac{\hbar^2}{4\mu q^2} \frac{\alpha(k, \kappa)}{L^4(k, \kappa)} \tag{33}$$

to get an expression for C as a function of z_0 close to zero, with a limit of 1.25 when $z_0 \to 0$, as shown in Fig. S5.

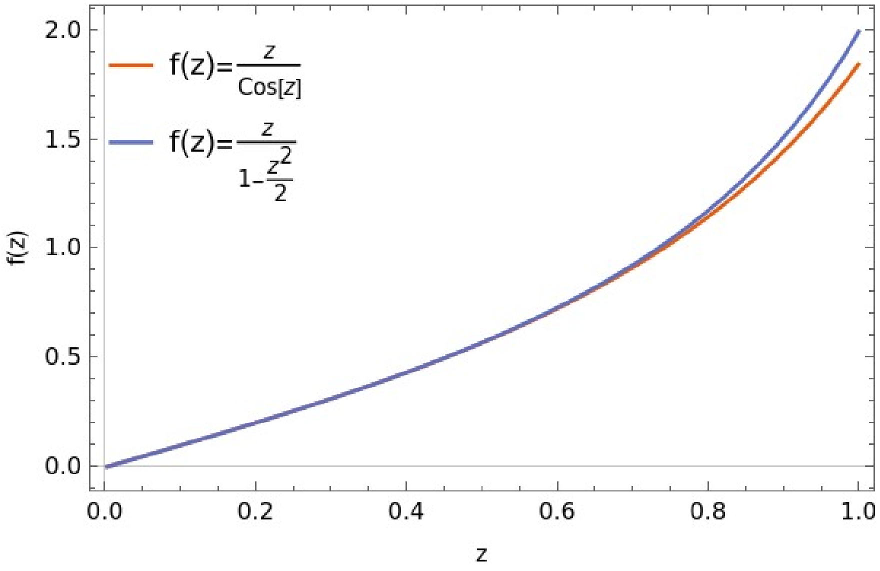

Figure S4: The function $z/\cos(z)$ is approximated with $z/(1-z^2/2)$ for small values of z in the limit $z_0 \to 0$.

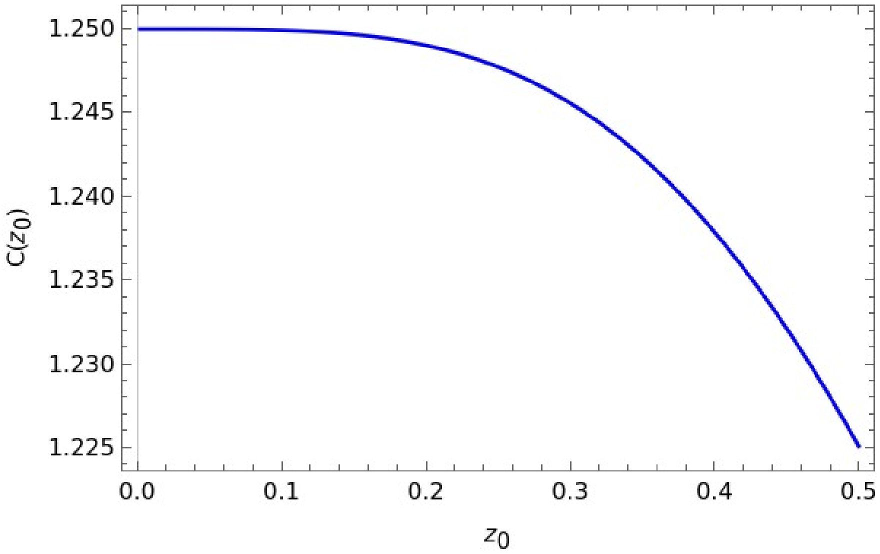

Figure S5: C coefficient for a particle bound in a finite square well potential at the limit $z_0 \to 0$.

Appendix D.

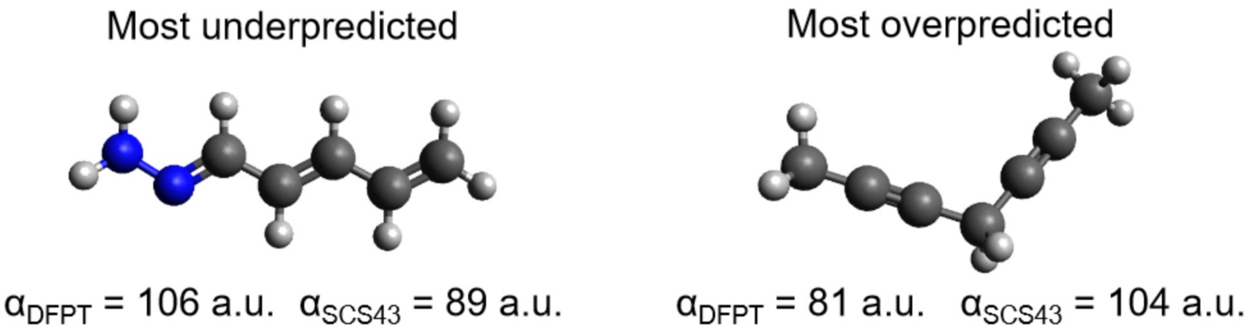

Figure S6: The most overpredicted and most underpredicted polarizability in the QM7–X dataset using our SCS43 approach.
